# Biographical

**Published:** 1876-03

**Authors:** 


### BIOGRAPHICAL.

Dr. Harris Ralph Smith was born in the town of Brownington, Orleans Co., Vermont, on the 2d day of February, 1821.

The son of a farmer of good family, but of moderate means. Of a nervous, energetic, enthusiastic temperament; of a warm-hearted and ambitious disposition; he was the pet of a large family and ever among the foremost in his classes, and clans at school. Although of a delicate constitution from early age, he was ever ready to battle lustily, either physically, mentally or morally, in any cause in which his impulse or sense of justice and propriety caused him to enlist. Among his boy associates and play mates, he would fight for whom he believed to be innocent and right, as quick and earnestly as in after life; he would contend for his position, though the united world were against him. He was a positive character. He loved his friends to the point of sacrificing his life for them, and he hated his enemies to the verge of exterminating warfare. Yet withal he was not vindictive and would never assail a foe unless provoked to the encounter. This was his disposition professionally as well as socially. As a consequence his enemies were bitter, his friends warm and true.

These characteristics clung to him through his life.

His education commenced in the common district school, was continued through the full academic course, under the preceptorship of Rev. A. L. Twilight, one of the first and ablest instructors in New England. On leaving the academy he was prepared to enter college two years in advance.

After the age of 17 years he had employed most of the vacations in teaching and the study of medicine; which he had early determined to adopt as his profession. As a teacher he was very successful, being rigidly and exactingly practical in everything he undertook, never advancing any theory unless he was prepared to demonstrate—at least, its feasibility.

In the fall of 1844 he formed a sudden determination of abandoning the literary college course and entering at once upon the prosecution of his professional studies. At that time his medical preceptor, Dr. E. Robinson, a brother-in-law, a skillful physician and surgeon, was about to remove to Wisconsin to engage in practice; Harris concluded to go with him. They went to the town of Southport, (now Kenosha.) There continuing his study of medicine he soon entered the office of Dr. Powers, dentist, as a student; no doubt induced thereto by the new impetus about that time given to the specialty of dentistry, by which it assumed an honorable rank among the scientific professions, through the noble exertions of such men as C. A. Harris and his compeers.

At the commencement of the lecture season he went to Chicago and matriculated for the course in "Rush Medical College." The course completed, he returned to Southport and resumed the study of dentistry, when after a few months his preceptor pronounced him fully competent as an operator. Supplying himself with a "kit" and a small stock of foil, teeth, etc., he started out as a practitioner, with good professional and varied pecuniary success,—itinerating through lower Wisconsin, Illinois and Indiana—opening an office for several weeks at a time in the larger towns, till the winter of 1845-6 he arrived at Terre Haute, Indiana, where he determined to locate, and opened a permanent office. For years his practice increased with the growth of that enterprising little city—then of only 4,000 inhabitants—and amounted to almost a monopoly.

Having been called to the chair of mechanical dentistry in the Ohio Dental College in 1854-5, he filled that position for several successive sessions, resuming his practice during vacations.

In May, 1857 he relinquished his practice in Terre Haute, to his partner and brother, S. B. Smith, and moved permanently to Cincinnati, where he entered into copartnership with Dr. S. L. Hamlen at No. 3 W. Fourth Street.

In the spring of 1862 he dissolved partnership with Hamlen and moved to No. 13 W. Fourth Street and in April 1872 moved to No. 80 W. Seventh Street, where he continued to practice his profession till his death, which occurred Dec. 30th, 1875.

From the very incipiency of his professional career he had ever a strong predilection for the practice of general medicine, and in the winter of '54 and '55 he, in connection with his duties as lecturer in the dental college, attended a course of medical lectures in the Ohio Medical College and graduated with fair honors, receiving the Degree and Diploma of M. D. Until some time subsequent to this he had not relinquished the idea of the practice of general medicine, but becoming convinced that his delicate constitution would not permit him to encounter the physical exposure consequent upon the life of the physician, he, with great reluctance, gave up the profession of medicine in its comprehensive sense, and settled down to the specialty of his choice—dentistry. In this he assumed and maintained an honorable position among his compeers—the *Orthodox* of the profession in Cincinnati.

If his prices were not always up to a "recherche" standard, his operations were sterling and honest. He never would consent to do inferior work for a cheap price. It was always a principle with him to do the best he could in every case, irrespective of what remuneration he would gain thereby. He was not a judicious *financeir*. The extent of this practice and the character of his operations should have rendered him a rich man long before the close of his labors, but, although his habits and those of his family were ever frugal and temperate, almost to the extreme, of economy, yet he left but a small accumulation of worldly goods.

He inherited a scorbutic diathesis from his maternal ancestor. All his life he had been, a so called, dyspeptic. The peculiar confinement and constrained position of the dental operator no doubt conspired to hasten and develop what proved to be a schirrous or cancerous affection of the stomach, assuming the form, as diagnosed by his physicians, of a general ulceration of the mucous membrane of the stomach, together with implication of the left lung, which some years ago had been injured by the rupture of a blood vessel, and from which he had experienced repeated hæmorrhages.

A year ago last September, he was constrained to give his practice into the hands of his brother, and for over one year he was an almost hopeless invalid, without any reasonable expectation of his recovery. In June, however, he was removed to his country-seat (at Loveland, 25 miles from the city) where he seemed to improve and gave some hopes to his friends of his final recovery. And in September he returned and assumed charge of his office, but was able to meet only a small share of the requirements of his patients.

On Christmas he was unable to eat his holiday dinner with the family, and from that time was confined to his bed, till the morning of Dec. 30, 1875, after a light breakfast, he was attacked with a hæmorrhage, (probably from the stomach) which proved fatal in less than an hour from the time of attack.

